# Early-phase laparoscopic skill acquisition in veterinary students: impact of 2D/3D vision and conventional/articulating instruments

**DOI:** 10.1186/s13620-025-00322-2

**Published:** 2025-12-19

**Authors:** Jongchul Yun, Sungin Lee

**Affiliations:** Department of Veterinary Surgery, College of Veterinary Medicine, Chungbuk National University, Chungdae-ro 1, Seowon-gu, Cheongju, 28644 Republic of Korea

**Keywords:** Laparoscopy, Laparoscopic training, Veterinary education, Pi score, Articulating laparoscopic instruments

## Abstract

**Background:**

Minimally invasive surgery has been increasingly adopted for its advantages of reduced postoperative pain and faster recovery, underscoring the importance of structured basic skills training. In human medicine, robotic surgery addresses the limitations of conventional laparoscopy, but widespread adoption in veterinary practice is constrained by cost and logistics. The potential for cost-effective alternatives such as three-dimensional (3D) visualization and novel articulating laparoscopic instruments (ALI) is growing, but evidence for their impact on novice training remains limited. This study investigated the influence of visualization systems (two-dimensional (2D) vs. 3D) and instrument types (conventional laparoscopic instruments (CLI) vs. ALI) on early-phase laparoscopic skill acquisition in veterinary students, and to assess feasibility of the performance improvement (Pi) score as an integrated metric.

**Results:**

Forty-eight students with no prior laparoscopic experience were randomized into four groups: 2D + CLI, 2D + ALI, 3D + CLI, and 3D + ALI (*n* = 12 per group). All the participants completed five repetitions of three standardized tasks on a box trainer (peg transfer, pattern cutting, needle guidance). The Pi scores were significantly positively correlated reductions in completion time (ρ = 0.443–0.674, *p* ≤ 0.002) and errors (ρ = 0.538–0.851, *p* < 0.001) across tasks. Compared with the ALI group, CLI groups achieved significantly greater performance improvements in peg transfer (F(1,44) = 5.251, *p* = 0.027; partial η² = 0.107) and needle guidance (F(1,44) = 9.435, *p* = 0.004; partial η² = 0.177). Although the main effect of vision was not significant, 3D group showed a consistent trend toward higher mean Pi scores than 2D group in all tasks (peg transfer: 1.384 vs. 1.338; pattern cutting: 1.350 vs. 1.311; needle guidance: 1.393 vs. 1.343). No interaction effects were detected. The Simulator Sickness Questionnaire and NASA Task Load Index scores did not differ between the groups.

**Conclusions:**

The Pi score provides a valid, comprehensive index reflecting both time and error in early-phase of laparoscopic training, enabling grade-based feedback. Compared with the ALI, the CLI showed higher short-term performance improvement in peg transfer and needle guidance. 3D vision has the potential to alleviate visuomotor demands without inducing simulator sickness. These findings suggest that laparoscopic training should be adapted to individual performance levels, learning objectives, and equipment-specific demands.

## Background

Laparoscopic surgery in veterinary medicine has rapidly advanced over the past two decades and has become widely used for minimally invasive approaches associated with reduced postoperative pain, faster patient recovery, and improved comfort compared to traditional open surgery [1–3]. Despite these clinical advantages, laparoscopic procedures require technical complexity and a learning curve for the surgeons due to intrinsic limitations, including two-dimensional (2D) vision, reduced tactile feedback, limited degrees of freedom of conventional laparoscopic instruments (CLI), and separation of visual and motor axes [4].

In human medicine, robotic surgical platforms such as the da Vinci system have been introduced to address these limitations. This system integrates three-dimensional (3D) vision to enhance depth perception and wristed instruments with over 360° of articulation to improve dexterity and precision for surgical outcomes [5]. However, their adoption in veterinary practice has been limited by high costs, prolonged operation times, large equipment sizes relative to small animal patients, and the need for substantial operating room space [6].

3D visualization systems have been shown to overcome the limitations of 2D vision by providing stereopsis, thereby enhancing depth perception, spatial orientation, and surgical precision. These advantages have been documented in basic laparoscopic skill assessments, where 3D vision significantly improved task performance and was highly accepted [7]. In clinical practice, a systematic review and meta-analysis of 25 randomized controlled trials reported that 3D vision reduced operative time by an average of 8% with no increase in complications or morbidity [8].

Novel articulating laparoscopic instruments (ALI) were developed to replicate the multi-degree-of-freedom dexterity of robotic systems with 360° wristed movement at the tip of the instruments while maintaining the accessibility and lower cost of conventional laparoscopy [9]. In human medicine, clinical studies in laparoscopic gastrectomy have demonstrated that novel ALI can offer practical advantages such as faster postoperative recovery and a trend toward fewer complications [10, 11]. However, their effectiveness in novice laparoscopic training remains underexplored, particularly in the context of structured laparoscopic training.

In the early stages of veterinary MIS, surgeons had limited opportunities for structured training and often transitioned rapidly into live surgery after only brief introductory courses [12]. Such approaches are considered unacceptable because of patient safety concerns and the growing availability of training options [13]. Therefore, simulation-based and structured curricula have become essential to ensure safe skill acquisition before clinical application.

Although the adoption of laparoscopic surgery and technological advances has increased in veterinary medicine, there is still a lack of evidence on how these modalities affect the acquisition of basic laparoscopic skills by novices. Therefore, this study aimed to evaluate the differences in early-stage laparoscopic performance improvement in basic laparoscopic skill according to imaging system (2D vs. 3D) and instrument (CLI vs. novel ALI; Artisential^®^) under time-constrained training conditions, and to evaluate the feasibility of the Pi score as an integrated metric for quantifying performance improvement.

## Methods

### Participants

Forty-eight veterinary medical students voluntarily participated in this study. All participants had normal or corrected-to-normal vision and no prior experience with laparoscopic procedures or structured laparoscopic skills training curricula. Prior to training, the questionnaire was completed by each participant regarding gender, age, handedness, academic year (3rd/4th), interest in surgery, surgery observation count, and experience performing a variety of activities that could influence hand–eye coordination (e.g., video games).

The participants were randomly assigned to one of four experimental groups (*n* = 12 per group) via computer-generated random allocation: Group 1: 2D vision + CLI; Group 2: 2D vision + ALI; Group 3: 3D vision + CLI; and Group 4: 3D vision + ALI.

### Instrument setup

**Fig. 1:**
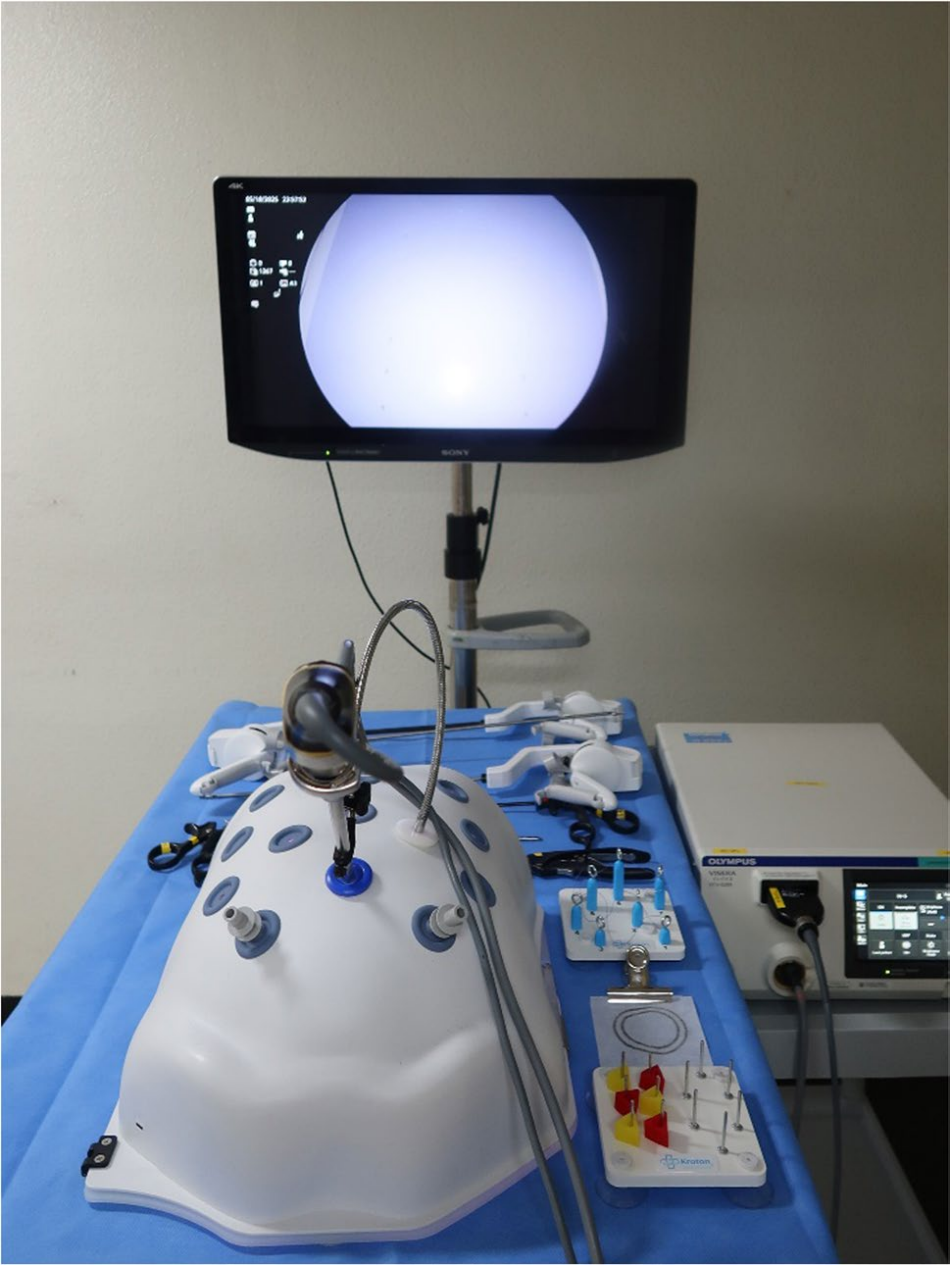
Experimental setup of the laparoscopic box trainer session. A video system center was used with either a 10-mm 30° rigid telescope for 2D or a 10-mm articulating stereoscopic videoscope for 3D. The task modules for peg transfer, pattern cutting, and needle guidance are shown

**Fig. 2:**
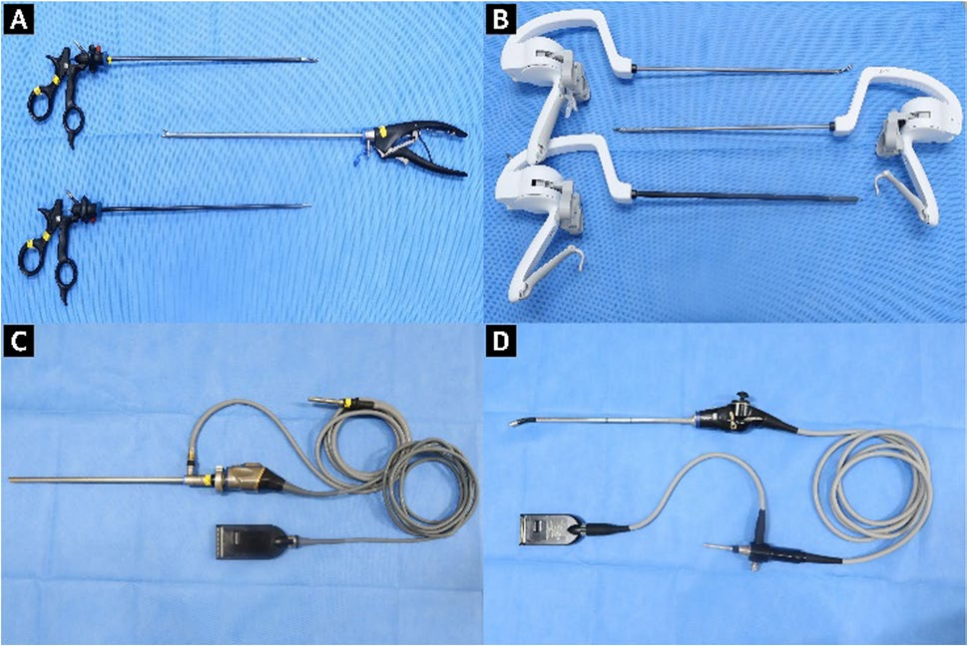
Instruments and scopes. **A** Conventional laparoscopic instruments (grasper, scissors, needle holders), (**B**) Articulating laparoscopic instruments (Artisential fenestrated forceps, monopolar scissors, and needle holders), (**C**) 2D system; 10-mm 30° rigid telescope with camera head, and (**D**) 3D system; articulating stereoscopic videoscope

A standardized box trainer model was used for all sessions to simulate the operative conditions (Fig. 1). Laparoscopic vision and LED light source were provided by an OTV-S300 video system center (Olympus, PA, USA). A 10-mm rigid telescope with 30° optics (Olympus, PA, USA) was used for 2D vision, whereas a 10-mm articulating videoscope (Endoeye^®^ Flex LTF-190; Olympus, PA, USA) was used for 3D vision (Fig. 2). The scope was fixed in a holder and adjusted to participant preference. The monitor was positioned at or slightly below the eye level. The participants in the 3D groups wore stereoscopic glasses.

CLI consisted of two grasping forceps, scissors and a needle holder (Olympus, PA, USA). The ALI consisted of two fenestrated forceps, monopolar scissors, and a needle holder (Livsmed, Seongnam, Korea). The placement of task modules and camera distance were kept constant to ensure uniform conditions for all participants.

### Procedure

All procedures were conducted in the veterinary surgery laboratory at Chungbuk National University. Before the training sessions, the participants were given a tutorial video for the correct performance of each task and a 20-minute acclimatization period to freely manipulate the assigned instruments.

**Fig. 3:**
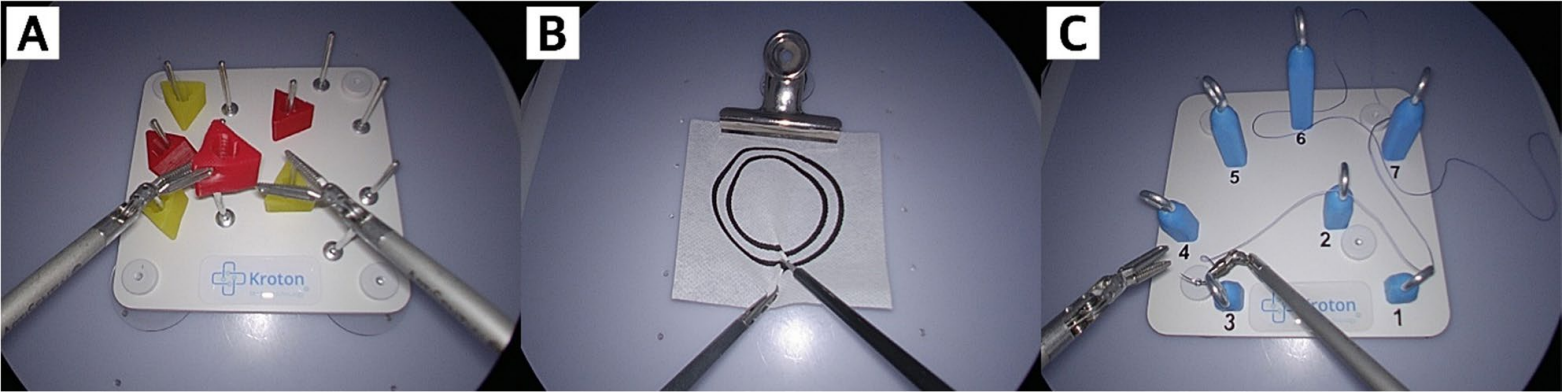
Endoscopic views of the three standardized tasks. **A** Peg transfer, (**B**) Pattern cutting, and (**C**) Needle guidance

In the training session, the participants performed five consecutive repetitions of the three standardized laparoscopic tasks (Kroton Medical Technology, Warsaw, Poland) in the same fixed order (Fig. 3). Five repetitions were chosen based on prior studies [14–16]. Three laparoscopic tasks, which are based on the European Training in Basic Laparoscopic Urological Skills (E-BLUS) curriculum, include peg transfer, pattern cutting, and needle guidance [17]. These tasks aim to train bimanual dexterity, hand-eye coordination, spatial awareness, and cutting skills.

#### Peg transfer

Participants were required to transfer six objects from one side of the pegboard to the other using two laparoscopic graspers. Each object was lifted with the nondominant hand, passed in mid-air to the dominant hand, then placed onto a peg on the opposite side. After all the objects were transferred, the procedure was reversed. Task duration was recorded from initial contact with the first object to the placement of the last object. Any dropped object was counted as an error.

#### Pattern cutting

Participants were required to incise a circle between two pre-marked concentric lines on a 4 × 4 inch piece of gauze using laparoscopic scissors and grasper. The gauze was suspended and secured to maintain constant tension. Task duration was recorded from the initial cut to the completion of the circle. Any deviation beyond the inner or outer line was counted as an error.

#### Needle guidance

Participants were required to pass a curved needle sequentially through the metal rings positioned along a predefined path as indicated by numbers using laparoscopic needle holder and grasper. Task duration was recorded from the initial entry of the needle into the first ring to its passage through the final ring. If the needle was dropped, it counted as an error. The measurement of task time resumed from the moment the needle was picked up.

For each repetition, the time to completion and number of errors were recorded. The performance improvement score (Pi score) was calculated according to the previously described formula: $\:Pi\:score=10*(\surd\:\frac{T1}{T2}*\frac{Tt}{T2}+\surd\:\frac{E1+1}{E2+1}*\frac{Et+1}{E2+1})$; T_1_: first trial time, T_2_: fifth trial time, T_t_: target time, E_1_: errors in first trial time, E_2_: errors in fifth trial, E_t_: target number of errors (set to 0) [14]. The target time is the cut-off time defined for the E-BLUS task; 126 s for peg transfer, 151 s for pattern cutting, and 268 s for needle guidance [14]. Data collection was recorded by the same investigator under consistent scoring criteria.

### Simulator comfort and workload assessment

Following the training session, participants completed both the Simulator Sickness Questionnaire (SSQ) and the NASA Task Load Index (NASA-TLX) to assess subjective comfort and perceived workload.

The SSQ, a validated tool designed to evaluate symptoms associated with simulator use [18], was used to investigate the impact of different visualizations and instruments on physical and visual comfort. The SSQ consists of 16 symptoms rated on a 4-point scale (0 = none, 1 = slight, 2 = moderate, 3 = severe). Subscale scores for oculomotor, disorientation, and nausea were calculated via the weighted method, and total SSQ score was computed as the weighted subscale scores multiplied by a scaling factor [18].

The NASA-TLX is widely used as the gold standard for workload assessment by measuring six subscales (mental demand, physical demand, temporal demand, performance, effort, and frustration) [19, 20]. The total NASA-TLX score was calculated as the average of the six subscale scores [20].

### Statistical analysis

Baseline demographic variables were compared among the groups. The Shapiro-Wilk test was used to assess normality. Group comparisons for non-normally distributed continuous variables were performed using the Kruskal-Wallis test, while categorical variables were compared using the chi-square test.

For each task, descriptive statistics of the Pi scores were calculated for each group. The changes in time (ΔTime) and errors (ΔError) were defined as the differences between the first (Trial 1) and the fifth trial (Trial 5) for each task. Spearman’s rank correlation coefficients (ρ) were calculated to evaluate the associations between Pi scores and both ΔTime and ΔError. On the basis of grades from the previous study, Pi scores were categorized into four grades (low, average, excellent, outstanding) for each task [14].

For nonnormally distributed data of Pi scores, logarithmic transformation was performed prior to analysis [21]. Two-way ANOVA of the Pi scores with vision (2D vs. 3D) and instrument (CLI vs. ALI) as factors was performed with log-transformed data to assess the main and interaction effects.

For the comparison of the SSQ and NASA-TLX scores, the Kruskal-Wallis test was applied. Statistical significance was set at *p* < 0.05. All the statistical analyses were performed using SPSS Statistics version 27.0 (IBM Corp., Armonk, NY, USA) and GraphPad Prism version 9.0 (GraphPad Software, San Diego, CA, USA).

## Results

### Demographic data

A total of 48 participants were included in the study. The baseline demographic characteristics of the participants are summarized in Table 1. Statistical analysis revealed no significant differences among the four groups.

**Table 1:** Participant characteristics

Variable	2D + CLI (*n* = 12)	2D + ALI (*n* = 12)	3D + CLI (*n* = 12)	3D + ALI (*n* = 12)	*p*-value
Gender (n = male: female)	4/8	5/7	5/7	6/6	0.877
Age (mean ± SD)	25.33 ± 3.20	24.08 ± 1.24	24.25 ± 1.91	25.50 ± 2.84	0.531
Academic year (*n* = 3rd/4th)	6/6	5/7	7/5	5/7	0.821
Handedness (n = right/left)	10/2	12/0	9/3	11/1	0.283
Interest in surgery (low “1” – high “5”, Mean ± SD)	4.17 ± 0.83	4.25 ± 0.97	4.33 ± 0.78	4.17 ± 3.07	0.839
Number of surgeries observed (mean ± SD)	5.83 ± 6.97	5.33 ± 3.89	5.33 ± 4.31	4.17 ± 3.07	0.928
Hand-eye coordination activities (n = yes/no)	10/2	8/4	7/5	9/3	0.569

Data is presented as mean ± standard deviation (SD) for continuous variables and as counts for categorical variables. No statistically significant differences were observed among groups for any baseline variable (all *p* > 0.05)

### Performance improvement

Pi scores showed significant positive correlations with both ΔTime and ΔError across all tasks (Table 2). For peg transfer, the correlations were ρ = 0.674 (*p* < 0.001) with ΔTime and ρ = 0.724 (*p* < 0.001) with ΔError. For pattern cutting, the correlations were ρ = 0.443 (*p* = 0.002) with ΔTime and ρ = 0.851 (*p* < 0.001) with ΔError. For needle guidance, the correlations were ρ = 0.472 (*p* = 0.001) with ΔTime and ρ = 0.538 (*p* < 0.001) with ΔError. These results indicate that Pi scores were significantly related to improvements in both time and error.

**Table 2:** Correlation between Pi scores and ΔTime/ΔError

Task	ΔTime (mean ± SD)	ΔError (mean ± SD)	ΔTime vs. Pi score (ρ, *p*)	ΔError vs. Pi score (ρ, *p*)
Peg transfer	8.833 ± 10.599	0.770 ± 0.881	0.674, < 0.001	0.724, < 0.001
Pattern cutting	5.041 ± 10.340	1.020 ± 1.228	0.443, 0.002	0.851, < 0.001
Needle guidance	23.666 ± 40.375	0.645 ± 1.041	0.472, 0.001	0.538, < 0.001

Values represent Spearman’s rank correlation coefficients (ρ) with corresponding p-values. ΔTime was defined as the difference between the first and fifth trial completion time, and ΔError as the difference between the first and fifth trial error counts. All three tasks demonstrated significant positive correlations between Pi scores and both ΔTime and ΔError (*p* < 0.05), supporting the construct validity of the Pi score as an integrated performance metric.

For peg transfer, the mean log-transformed Pi score was 1.338 ± 0.095 for the 2D vision group and 1.384 ± 0.082 for the 3D vision group. By instrument type, the mean log-transformed Pi score was 1.389 ± 0.084 for the CLI group and 1.333 ± 0.090 for the ALI group. No significant interaction was observed between vision and instrument type (F(1,44) = 0.416, *p* = 0.522, partial η² = 0.009). There was a statistically significant main effect of instrument type (F(1,44) = 5.251, *p* = 0.027, partial η² = 0.107), with the CLI group showing higher performance improvements compared to ALI groups. The main effect of vision revealed a tendency but did not reach statistical significance (F(1,44) = 3.424, *p* = 0.071, partial η² = 0.072).

For pattern cutting, the mean log-transformed Pi score was 1.311 ± 0.106 for the 2D vision group and 1.350 ± 0.115 for the 3D vision group. By instrument type, the mean log-transformed Pi score was 1.345 ± 0.109 for the CLI group and 1.321 ± 0.114 for the ALI group. No significant interaction was observed between vision and instrument type (F(1,44) = 0.011, *p* = 0.918, partial η² = 0). Neither the main effect of vision (F(1,44) = 1.105, *p* = 0.299, partial η² = 0.12) nor instrument type (F(1,44) = 0.520, *p* = 0.475, partial η² = 0.24) revealed statistical significance.

For needle guidance, the mean log-transformed Pi score was 1.343 ± 0.136 for the 2D vision group and 1.393 ± 0.125 for the 3D vision group. By instrument type, the mean log-transformed Pi score was 1.421 ± 0.140 for the CLI group and 1.314 ± 0.098 for the ALI group. No significant interaction was observed between vision and instrument type (F(1,44) = 0.063, *p* = 0.804, partial η² = 0.001). There was a statistically significant main effect of instrument type (F(1,44) = 9.435, *p* = 0.004, partial η² = 0.177) with the CLI group showing higher performance improvements compared to ALI groups.

**Fig. 4:**
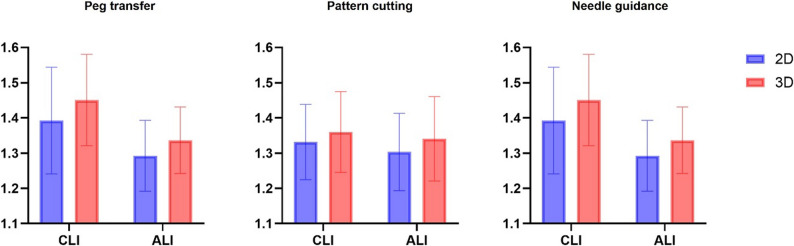
Two-way ANOVA results for log-transformed Pi scores (mean ± SD) across vision (2D vs. 3D) and instrument type (CLI vs. ALI). The error bars indicate the SD. The instrument showed a significant main effect on peg transfer (*p* = 0.027) and needle guidance (*p* = 0.004); vision had no significant main effect across tasks; no interaction effects were detected

**Fig. 5:**
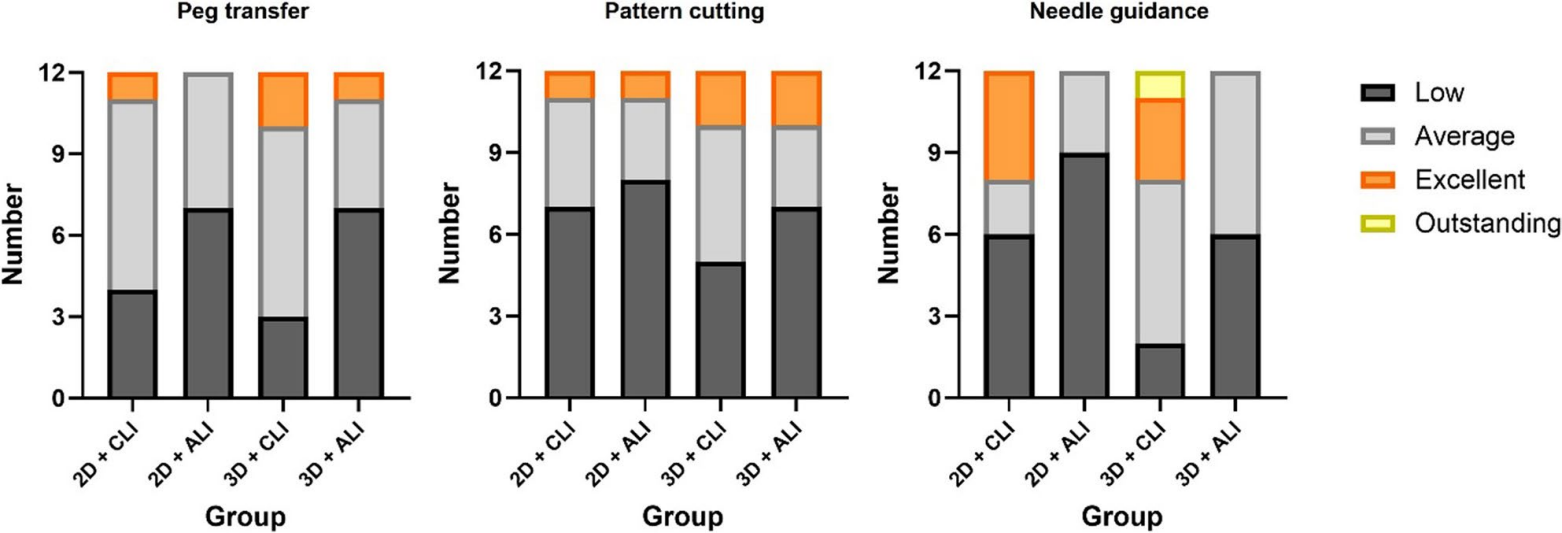
Distribution of Pi score-based performance grades by group. Stacked bars show the number of students classified as low, average, excellent, or outstanding for each task; grades were based on predefined cut-offs

The overall comparisons of Pi scores for the three tasks are presented in Tables 3 and 4, with the two-way ANOVA results illustrated in Fig. 4. The distribution of Pi grades, which is based on predefined criteria from a previous study, is illustrated in Fig. 5 [14].

**Table 3:** Log-transformed Pi scores of three laparoscopic tasks

Task	Group 1 (2D + CLI)	Group 2 (2D + ALI)	Group 3 (3D + CLI)	Group 4 (3D + ALI)	Main effect; vision (*p*)	Main effect; instrument (*p*)	Interaction (*p*)
Peg transfer (Log, mean ± SD)	1.359 ± 0.096	1.318 ± 0.094	1.421 ± 0.060	1.348 ± 0.088	0.071	0.027*	0.522
Pattern cutting (Log, mean ± SD)	1.330 ± 0.106	1.303 ± 0.110	1.360 ± 0.115	1.340 ± 0.120	0299	0.475	0.918
Needle guidance (Log, mean ± SD)	1.392 ± 0.151	1.293 ± 0.103	1.451 ± 0.129	1.335 ± 0.095	0.083	0.004*	0.804

Values are presented as mean ± standard deviation (SD) of log-transformed Pi scores. Statistical analysis was performed using two-way ANOVA with factors vision (2D vs. 3D) and instrument (CLI vs. ALI). *p* < 0.05 indicates statistical significance

**Table 4:** Raw Pi scores of three laparoscopic tasks

Task	Group 1 (2D + CLI)	Group 2 (2D + ALI)	Group 3 (3D + CLI)	Group 4 (3D + ALI)
Peg transfer (Raw, median [IQR])	24.14 [6.72]	19.33 [8.32]	27.10 [6.63]	21.29 [6.00]
Pattern cutting (Raw, median [IQR])	21.78 [10.24]	18.66 [10.84]	24.35 [9.74]	20.66 [11.72]
Needle guidance (Raw, median [IQR])	24.53 [16.36]	19.33 [8.07]	27.77 [12.92]	22.89 [7.60]

Values are presented as median with interquartile range (IQR) of raw Pi scores. These results are provided as supplementary information to complement the main analysis

#### Simulator comfort and workload

No statistically significant differences in the total SSQ socres were identified among the four groups (Table 5). Median total SSQ scores (IQR, mean ± SD) were 1.82 (3.71, 2.66 ± 2.52), 2.30 (2.67, 2.56 ± 1.73), 0.81 (2.27, 1.35 ± 1.17), and 2.32 (2.76, 2.76 ± 2.20) for each group.

NASA-TLX scores for subjective workload assessment did not differ significantly between the groups (Table 5). The median scores (IQR, mean ± SD) were 53.33 (20.83, 53.05 ± 14.81), 54.16 (22.92, 52.77 ± 12.81), 53.30 (21.25, 48.19 ± 12.09), and 49.16 (20.83, 52.08 ± 12.39) for each group.

**Table 5:** Simulator comfort and workload

Variable	Group 1 (2D + CLI)	Group 2 (2D + ALI)	Group 3 (3D + CLI)	Group 4 (3D + ALI)	*p*-value
SSQ scores (median [IQR])	1.82 [3.71]	2.30 [2.67]	0.81 [2.27]	2.32 [2.76]	0.148
NASA-TLX (median [IQR])	53.33 [20.83]	54.16 [22.92]	53.3 [21.25]	49.16 [20.83]	0.783

Values are presented as median [interquartile range, IQR]

*SSQ* Simulator Sickness Questionnaire, *NASA-TLX* National Aeronautics and Space Administration Task Load Index

No statistically significant differences were observed among groups for either SSQ or NASA-TLX scores (all *p* > 0.05)

## Discussion

MIS has been increasingly applied in veterinary surgical practice with the aim of reducing postoperative pain and shortening the time required for patients to normal activity [22]. Indeed, a recent survey of 139 small animal diplomate and resident veterinary surgeons in the United States reported a substantial increase in soft tissue MIS caseloads [23]. As MIS caseloads continue to expand, the incorporation of structured MIS training remains a critical component in the preparation of veterinary surgeons for clinical practice.

The structured curricula for basic laparoscopic skills have demonstrated that simulator-based assessment can provide reliable standardized assessment frameworks. In a prospective study of 40 surgical trainees, FLS scores were significantly correlated with intraoperative performance as measured by the Global Operative Assessment of Laparoscopic Skills (*r* = 0.77, *p* < 0.001) [24]. Similarly, the E-BLUS curriculum defines standardized tasks, target times, and objective pass/fail criteria, which support reproducible assessment and serve as a common framework for multicenter comparison [25]. In this study, the protocol was aligned with E-BLUS via three tasks and Pi scores in reference to E-BLUS cut-off value, enhancing cross-study comparability and reproducibility.

Simulator-based assessments have generally relied on separate measurements of time and error, with performance being disproportionately influenced by completion time. As the chronometric pressure increases, the performance accuracy tends to decrease, which may result in an inaccurate assessment of basic skill acquisition [26]. The Pi score has been proposed as an integrated measure that combines time and error into a single index, providing a quantitative evaluation of basic laparoscopic skill acquisition [14]. This scoring system is designed to align performance evaluations more closely with surgical competency requirements by emphasizing that rapid completion of tasks is not sufficient unless accuracy is ensured [14]. In the present study, the Pi score was effective in identifying differences between groups. This validity was further supported by correlation analysis, which demonstrated that Pi scores were significantly correlated with both ΔTime (ρ = 0.443–0.674, *p* ≤ 0.002) and ΔError (ρ = 0.538–0.851, *p* < 0.001) across all tasks. These findings support its ability to accurately reflect the overall performance improvement of novices and to discriminate across experimental conditions. In addition, the Pi score offers the advantage of grading system allowing the classification of trainees’ performance levels into distinct categories for customized feedback.

Previous studies in laparoscopic novices have reported contradictory findings regarding the comparative advantages of CLI and ALI. While a pilot randomized study demonstrated that the use of motorized ALI improved task completion times [27], another randomized controlled trial reported that CLI was associated with significantly faster completion times and fewer failures than ALI [28]. In the present study, ALI was associated with significantly lower performance improvements in peg transfer and needle guidance than CLI. While CLI operates primarily through linear movements constrained by the fulcrum effect, ALI represents a distinct paradigm of motion control, closely mimicking the wristed articulation of robotic surgery with multiple degrees of freedom. This mechanism increases manipulation complexity, excessive wrist twisting and more deviated wrist posture [29, 30]. For novices, the fundamental difference can manifest as increased ergonomic load requiring greater coordination and adaptation to unconventional wrist postures. These findings suggest that ALI should be introduced after fundamental coordination with CLI has been achieved. A stepwise, proficiency-based progression from CLI to ALI may enable novices to establish stable motion control before adapting to the additional degrees required for ALI. Dedicated articulation training that emphasizes wrist articulation, instrument triangulation, and ergonomic adaptation could help alleviate manipulation complexity and improve precision.

Although potential advantages of 3D visualization for basic laparoscopic skill acquisition have been suggested [31, 32], the main effect of vision was not significant in this study. In basic tasks, depth and spatial relationships can be relatively easily reconstructed from monocular cues such as shading, relative size, object interposition, and texture gradient [33], which may limit the extent to which stereopsis translates into a group-level effect. In clinical settings, procedures can be performed using monocular depth cues, but these cues cannot completely replace stereopsis, resulting in increased operating time and reduced accuracy [33]. These findings are consistent with the observation that, although the main effect of vision was not significant, a consistent trend of higher mean Pi scores was observed under 3D vision across all tasks (Peg transfer: 3D = 1.384 vs. 2D = 1.338; Pattern cutting: 3D = 1.350 vs. 2D = 1.316; Needle guidance: 3D = 1.393 vs. 2D = 1.342). Moreover, no significant differences were identified in the SSQ scores between the groups. In this regard, 3D vision may have the potential to partially alleviate the load of visuomotor mapping in the initial phase of training without increasing simulator sickness. Therefore, the appropriate use of 3D vision during the initial phase of MIS training curricula can be an effective strategy to accelerate proficiency acquisition in basic laparoscopic tasks.

Pattern cutting is a fundamental task used to standardize the assessment of precision in laparoscopic surgery and is widely incorporated into training curricula, such as the FLS and the E-BLUS program owing to its essential role in surgical education and evaluation [17, 34]. In this study, unlike the peg transfer and needle guidance tasks, no significant differences between groups were detected in the pattern cutting task (*p* = 0.654). This finding aligns with prior studies that reported no significant differences in performance on this task across vision or instrument types, despite different outcomes in other tasks such as peg transfer or suturing [35–38]. This can be attributed to the inherently simple and repetitive nature of pattern cutting which primarily requires planar contour tracking with minimal demands on depth perception or instrument articulation. Thus, pattern cutting allows novices to focus on maintaining precision, tension control and hand-eye coordination without excessive cognitive load. Therefore, rather than a comparative tool, it may be more appropriate as an early-phase performance assessment task within structured training curricula.

Several limitations should be acknowledged when these findings are interpreted. First, the study focused on short-term training and immediate performance without long-term evaluation to assess skill retention or transferability to clinical settings. Second, the Pi score was effective in assessing performance during the initial training phase, but discriminative capacity may diminish as proficiency plateaus indicating that its applicability may be limited to early-stage training. Third, the limited sample size may affect the generalizability of the results. Fourth, as this study was conducted in a simulated training environment with a fixed number of repetitions, the outcomes may not fully represent performance under real surgical conditions or account for individual variability in learning curves. Finally, although three representative tasks were selected from the E-BLUS curriculum, more complex tasks such as intracorporeal suturing were not included. While previous studies have also relied on a subset of tasks, the omission of advanced tasks limits comprehensiveness and may underestimate the challenges associated with higher-level laparoscopic skill acquisition.

## Conclusion

In conclusion, this study demonstrated that the Pi score provides a balanced assessment of early-stage basic laparoscopic skill acquisition by integrating both time and error into a comprehensive index. Performance improvement was influenced by equipment type, with ALI imposing greater ergonomic and cognitive demands than CLI. Although not statistically significant, 3D vision has the potential to alleviate visuomotor demands in novices. These findings suggest that laparoscopic training should be adapted to individual performance levels, training objectives, and modality-specific demands.

## Data Availability

The datasets generated and analyzed during the current study are available from the corresponding author on reasonable request.
